# Fragilides K and L, New Briaranes from the Gorgonian Coral *Junceella fragilis*

**DOI:** 10.3390/molecules23071510

**Published:** 2018-06-22

**Authors:** Li-Guo Zheng, Yu-Chia Chang, Chiung-Chih Hu, Zhi-Hong Wen, Yang-Chang Wu, Ping-Jyun Sung

**Affiliations:** 1 Graduate Institute of Marine Biology, National Dong Hwa University, Pingtung 94450, Taiwan; t0919928409@gmail.com; 2 National Museum of Marine Biology and Aquarium, Pingtung 94450, Taiwan; jay0404@gmail.com (Y.-C.C.); smallsmallhu@gmail.com (C.-C.H.); 3 Research Center for Chinese Herbal Medicine, Research Center for Food and Cosmetic Safety, Graduate Institute of Health Industry Technology, College of Human Ecology, Chang Gung University of Science and Technology, Taoyuan 33303, Taiwan; 4 Department of Marine Biotechnology and Resources, National Sun Yat-sen University, Kaohsiung 80424, Taiwan; 5 Graduate Institute of Natural Products, Kaohsiung Medical University, Kaohsiung 80708, Taiwan; 6 Research Center for Natural Products and Drug Development, Kaohsiung Medical University, Kaohsiung 80708, Taiwan; 7 Department of Medical Research, Kaohsiung Medical University Hospital, Kaohsiung 80756, Taiwan; 8 Chinese Medicine Research and Development Center, China Medical University Hospital, Taichung 40447, Taiwan

**Keywords:** *Junceella fragilis*, briarane, fragilide, iNOS, COX-2

## Abstract

Two new briarane metabolites—fragilides K (**1**) and L (**2**)—along with five known analogues—gemmacolide X, praelolide, juncins P and ZI, and gemmacolide V (**3**–**7**)—were extracted and purified from *Junceella fragilis*, a gorgonian coral. Based on data obtained via spectroscopic techniques, the structures of new briaranes **1** and **2** were determined and the cyclohexane rings in **1** and ***2*** were found to exist in chair and twist boat conformation, respectively. Additionally, anti-inflammatory analysis showed that briaranes **2**, **3**, and **6** inhibited pro-inflammatory inducible nitric oxide synthase protein expression and briaranes **3** and **7** suppressed the cyclooxygenase-2 level, in LPS-stimulated murine macrophage-like RAW264.7 cells.

## 1. Introduction

**Figure 1:**
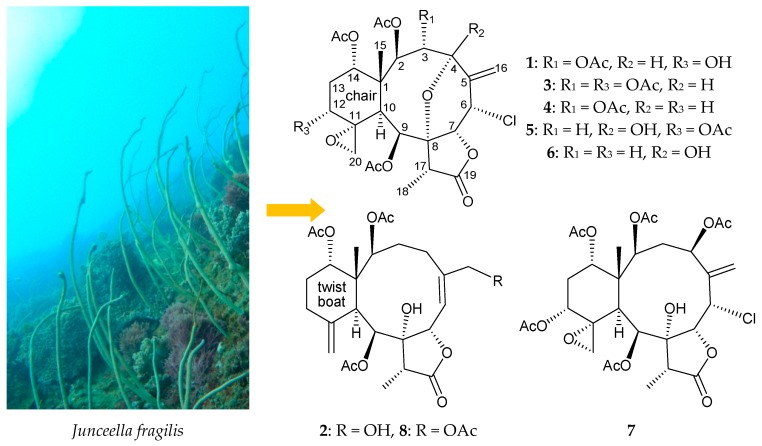
Gorgonian coral *Junceella fragilis* and structures of fragilides K (**1**) and L (**2**), gemmacolide X (**3**), praelolide (**4**), juncins P (**5**) and ZI (**6**), gemmacolide V (**7**), and junceellonoid B (**8**).

*Junceella fragilis* (Ridley, 1884) (family Ellisellidae) [1,2,3], a gorgonian coral, has been reported to contain high levels of diterpenoids with a briarane carbon skeleton. These diterpenoids often have complex structures and possess varied bioactivities [4,5,6,7,8,9]. In further studies of the chemical constituents of *J. fragilis*, two new briaranes—fragilides K (**1**) and L (**2**)—and five known analogues—gemmacolide X (**3**) [10], praelolide (**4**) [11,12,13,14,15,16], juncins P (**5**) and ZI (**6**) [16,17], and gemmacolide V (**7**) [10]—were obtained (Figure 1). In this study, we isolated and determined the structures of these briaranes, and performed anti-inflammatory assays to determine their anti-inflammatory activities in terms of targeting inducible nitric oxide synthase (iNOS) and cyclooxygenase-2 (COX-2) in a macrophage in vitro system.

## 2. Results and Discussion

Fragilide K (**1**), [α]${}_{D}^{22}$ –43 (*c* 0.02, CHCl_3_), was obtained as an amorphous powder. The molecular formula of **1** was established as C_28_H_35_ClO_13_ (11 degrees of unsaturation) from a sodiated molecule at *m*/*z* 637 in the electrospray ionization mass spectrum (ESIMS), and further supported by the high-resolution electrospray ionization mass spectrum (HRESIMS) at *m*/*z* 637.16613 (calcd. for C_28_H_35_ClO_13_ + Na, 637.16584). The IR spectrum of **1** demonstrated bands at 3545, 1790, and 1740 cm^–1^, which were consistent with the presence of hydroxy, γ-lactone, and ester carbonyl groups. The ^13^C NMR and distortionless enhancement by polarization transfer (DEPT) spectra showed that **1** had 28 carbons (Table 1): 6 methyls, 2 sp^3^ methylenes, 10 sp^3^ methines, 3 sp^3^ quaternary carbons, 1 sp^2^ methylene, and 6 sp^2^ quaternary carbons.

From the ^1^H and ^13^C NMR spectra (Table 1), **1** was found to possess four acetoxy groups (δ_H_ 2.32, 2.08, 2.05, 2.00, each 3H × s; δ_C_ 21.1, 21.1, 20.5, 20.3, acetate methyl × 4; δ_C_ 169.5, 169.9, 170.2, 169.9, acetate carbonyl × 4), a γ-lactone moiety (δ_C_ 174.2), and an exocyclic carbon–carbon double bond (δ_C_ 134.1, C; 119.7, CH_2_; δ_H_ 5.36, 1H, d, *J* = 1.6 Hz; 5.57, 1H, d, *J* = 1.6 Hz). Thus, based on the aforementioned data, six degrees of unsaturation were accounted for, and **1** was identified as a pentacyclic compound. The presence of an exocyclic epoxy group was confirmed from the signals of an oxygenated quaternary carbon at δ_C_ 58.9 (C) and an oxymethylene at δ_C_ 50.6 (CH_2_). The chemical shifts of oxymethylene protons at δ_H_ 2.76 (1H, d, *J* = 3.2 Hz) and 2.56 (1H, d, *J* = 3.2 Hz) supported the presence of this group. From the ^1^H–^1^H correlation spectroscopy (COSY) spectrum of **1**, four different structural units, H-2/H-3/H-4, H-6/H-7, H-12/H_2_-13/H-14, and H-17/H_3_-18, were identified (Table 1), which were assembled with the assistance of a heteronuclear multiple-bond coherence (HMBC) experiment (Table 1). The HMBC correlations between protons and quaternary carbons of **1**, such as H-2, H-3, H-9, H-10, H_3_-15/C-1; H-3, H-4, H-7/C-5; H-4, H-10, H_3_-18/C-8; H-9, H-10/C-11; and H-17, H_3_-18/C-19, permitted elucidation of the carbon skeleton of **1**. An exocyclic double bond at C-5 was confirmed by the HMBC correlations between H_2_-16/C-4, C-6. The ring junction C-15 methyl group was positioned at C-1 from the HMBC correlations between H_3_-15/C-1, C-2, C-10, C-14 and H-2, H-10/C-15. The acetoxy groups at C-2, C-3, and C-9 were established by correlations between H-2 (δ_H_ 5.50), H-3 (δ_H_ 6.22), H-9 (δ_H_ 5.60) and the acetate carbonyls at δ_C_ 170.2, 169.9, 169.5, observed in the HMBC spectrum of **1**. The remaining hydroxy group and acetate ester were positioned at C-12 and C-14, respectively, as indicated by characteristic ^1^H NMR signal analysis (δ_H_, 3.49, 1H, br s, H-12; 5.01, 1H, dd, *J* = 3.2, 3.2 Hz, H-14).

From the ESIMS spectrum [(M + Na)^+^:(M + 2 + Na)^+^ = 3:1], the intensity of the sodiated molecule isotope peak [M + 2 + Na]^+^ was noted, which confirmed the presence of a chlorine atom in **1**. The methine unit at δ_C_ 53.8 (CH) was more shielded than would be expected for an oxygenated C-atom and was correlated with the methine proton at δ_H_ 4.96 in the heteronuclear single-quantum coherence (HSQC) spectrum; this proton also showed a ^3^*J*-correlation with H-7 in the ^1^H–^1^H COSY spectrum, confirming the attachment of a chlorine atom at C-6. In addition, the methylene unit at δ_C_ 50.6 was correlated with the methylene protons at δ_H_ 2.76 and 2.56 in the HSQC spectrum, and the HMBC correlations between H-9, H-10/C-11 (an oxygenated quaternary carbon, δ_C_ 58.9), and H-10/C-20, confirmed the attachment of an epoxy group at C-11/20. Furthermore, an HMBC correlation between H-4 (δ_H_ 4.49) and an oxygenated quaternary carbon at δ_C_ 83.0 (C-8) suggested the presence of a C-4/8 ether linkage in **1**.

**Figure 2:**
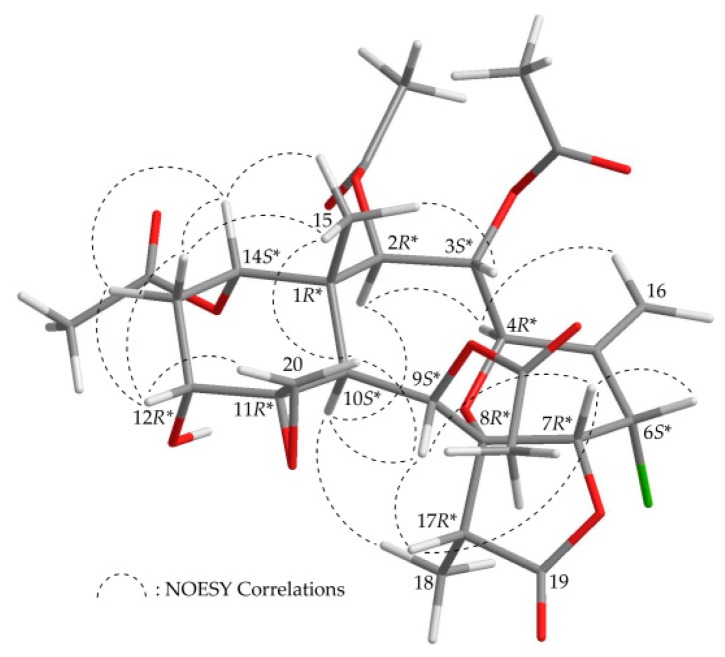
A model of **1** generated using a computer-assisted system based on the data from MM2 force field calculations and selected protons with key NOESY correlations.

From the findings of previous surveys, all briaranes that exist naturally have H-10 trans to a C-15 methyl, and these two groups are assigned as α- and β-oriented in most briarane analogues [18]. The relative stereochemistry of **1** was established from the interactions observed in a nuclear Overhauser effect spectroscopy (NOESY) experiment and by vicinal ^1^H–^1^H coupling constant analysis, which was corroborated by MM2 force field calculations (Figure 2) [19], indicating the most stable configuration to be as shown in Figure 2. The results of the NOESY experiment showed correlations between H-10 and H-2, H-9, and H_3_-18 in **1**, suggesting that these protons were situated on the same face of the molecule, and therefore they were assigned as α protons, as Me-15 was β-oriented and H_3_-15 did not show a correlation with H-10. The oxymethine protons H-3 and H-14, and one proton of the C-20 methylene (δ_H_ 2.56, H-20b), were found to exhibit interactions with H_3_-15, but not with H-10, revealing that H-3 and H-14 were β-oriented, and the epoxy group between C-11/20 was α-oriented. H-12 exhibited correlations with one proton of the C-20 methylene (δ_H_ 2.76, H-20a) and the C-13 methylene protons, but not with H-10, indicating that the hydroxy group at C-12 was α-oriented. H-9 was found to show correlations with H-7, H-10, H-17, and H-20b. From modeling analysis, H-9 was found to be reasonably close to H-7, H-10, H-17, and H-20b, and could therefore be placed on the α face in **1**, and methine protons H-7 and H-17 were β-oriented. H-7 showed correlations with H-6 and H-17, and a small coupling constant was found between H-6 and H-7 (*J* = 2.8 Hz), indicating that H-6 was of a β-orientation. Moreover, H-4 showed correlations with H-2 and one proton of the C-16 methylene (δ_H_ 5.36, H-16a), and a large coupling constant was found between H-3 and H-4 (*J* = 10.8 Hz), indicating that H-4 was of an α-orientation. The chemical shifts of the exocyclic 11,20-epoxy groups in briarane analogues have been summarized, and the ^13^C NMR shifts for C-11 and C-20 appear at δ_C_ 55–61 and 47–52 ppm, respectively, the epoxy group is α-oriented (11R*), and the cyclohexane ring is of a chair conformation [20]. Based on the above findings, the 11,20-epoxy group in **1** (δ_C_ 58.9, C-11; 50.6, CH_2_-20) was α-oriented, and the cyclohexane ring in **1** was of a chair conformation. Therefore, based on the above findings, the configurations of the stereogenic centers of **1** were elucidated as 1R*,2R*,3S*,4R*,6S*,7R*,8R*,9S*, 10S*,11R*,12R*,14S*, and 17R* (see Figures S1–S9 in the Supplementary Materials). 

Fragilide L (**2**) was obtained as an amorphous powder that gave an [M + Na]^+^ ion with *m*/*z* 531.22014 in the HRESIMS analysis, appropriate for the molecular formula C_26_H_36_O_10_ (calcd. for C_26_H_36_O_10_ + Na, 531.22007). Inspection of the IR spectrum revealed absorptions indicative of hydroxy (3447 cm^–1^), γ-lactone (1771 cm^–1^), and ester carbonyl (1731 cm^–1^) groups. From the ^1^H and ^13^C NMR data of **2** (Table 2), an exocyclic carbon–carbon double bond was deduced from the signals of two carbons at δ_C_ 150.9 (C) and 113.1 (CH_2_); this was further supported by two olefin protons at δ_H_ 5.06 (1H, s) and 4.90 (1H, s). Moreover, four carbonyl resonances at δ_C_ 175.8, 171.8, 170.6, and 169.4 confirmed the presence of a γ-lactone and three other ester groups. All the esters were identified as acetates by the presence of three acetyl methyl resonances in the ^1^H (δ_H_ 2.21, 2.01, and 1.91, each 3H × s) and ^13^C (δ_C_ 21.8, 21.1, and 21.2) NMR spectra (Table 2). It was found that the NMR data of **2** were similar to those of a known analogue, junceellonoid B (**8**) (Figure 1) [21], with the exception that the signals corresponding to the 16-acetoxy group in **8** were replaced by a hydroxy group in **2**. From the HMBC correlations, the presence of olefinic carbons at δ_C_ 146.7 (C) and 121.7 (CH), and oxygen-bearing methylene protons at δ_H_ 4.28 and 4.14, was noted, and a hydroxy group was deduced to be attached at C-16 in **2** (Table 2). The other HMBC correlations observed fully supported the locations of the functional groups, and hence fragilide L (**2**) was assigned with the structure of **2**, with the same stereochemistry as in junceellonoid B (**8**), because the stereogenic centers that **2** had in common with **8**, in addition to the ^1^H and ^13^C NMR chemical shifts and proton coupling constants matched well, and were further supported by a NOESY experiment.

**Figure 3:**
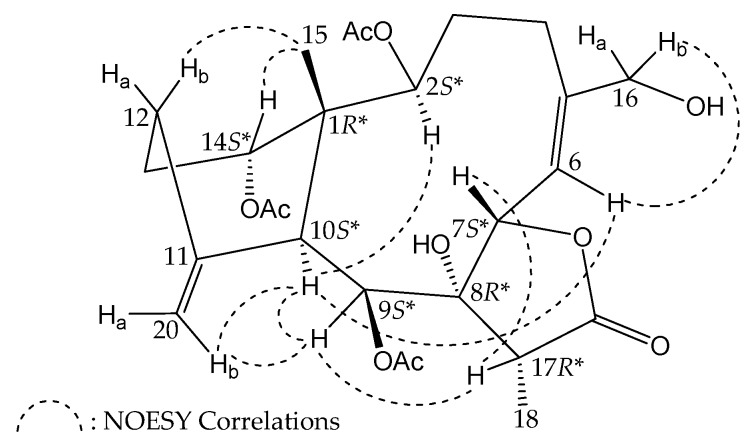
Selected protons with key NOESY correlations of **2**.

The relative stereochemistry of **2** was elucidated from the NOE interactions observed in a NOESY experiment (Figure 3). Due to the α-orientation of H-10, the ring junction C-15 methyl group is β-oriented, as no correlation was observed between H-10 and H_3_-15. In the NOESY spectrum of **2**, H-10 was correlated to H-2 and H-9, suggesting that H-2 and H-9 were located on the same face and can be assigned as α protons. H-14 was found to exhibit a response with H_3_-15, but not with H-10, showing that this proton was β-oriented. It was found that H-17 showed NOE correlations with H-7 and H-9. Consideration of molecular models revealed that H-17 is reasonably close H-7 and H-9 when H-7 and H-17 were β-oriented. The NOE correlations between H-6 and H-10 suggested that the Δ^5^ double bond in the 10-membered ring was oriented in such a way that the H-6 is on the same side as H-10. The large coupling constant observed (*J* = 10.0 Hz) revealed the antiparallel arrangement of H-6 and H-7 and the β orientation of H-7. H-6 exhibited a correlation with one proton of C-16 oxymethylene (δ_H_ 4.14, H-16b), suggesting the *Z*-configuration of C-5/6 double bond. Furthermore, a proton of the C-20 methylene (δ_H_ 5.06, H-20b) was found to exhibit NOE responses with H-9 and H-10, but not with H_3_-15, and H_3_-15 showed a NOE correlation with one proton of C-12 methylene (δ_H_ 2.18, H-12b), indicating the cyclohexane ring of **2** should be presented as a twist boat rather than a chair conformation for briarane **2**.

In a previous study, the proton chemical shifts of the briarane derivatives containing an 11,20-exocyclic carbon–carbon double were summarized and the difference between the two olefin protons (H-20a/b) was smaller than 0.2 ppm, whereas the cyclohexane rings exhibited a twisted boat conformation [22]. Owing to the chemical shifts of the C-20 methylene protons (δ_H_ 5.06 and 4.90), the configuration of the methylenecyclohexane ring in **2** was concluded to be of a twisted boat conformation. Based on the above findings, the configurations of the stereogenic centers of **2** were elucidated as 1R*,2S*,7S*,8R*,9S*,10S*,14S*, and 17R* (Supplementary Materials, Figures S10–S18).

Six known chlorinated briaranes were also isolated and were identified as gemmacolide X (**3**) [10], praelolide (**4**) [11,12,13,14,15,16], juncins P (**5**) and ZI (**6**) [16,17], and gemmacolide V (**7**) [10] by comparison with the spectroscopic and physical data reported in the literature.

**Figure 4:**
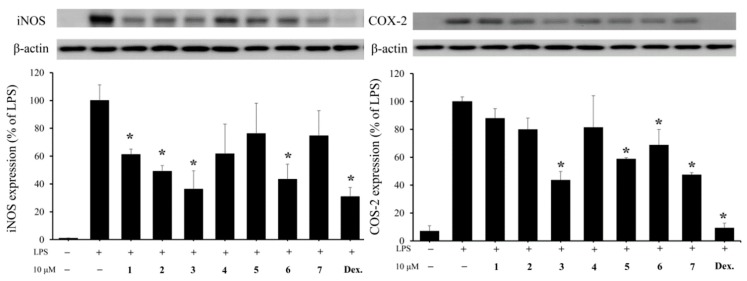
Effects of compounds **1**–**7** on the expression of pro-inflammatory iNOS and COX-2 proteins in lipopolysaccharide (LPS)-treated murine RAW264.7 macrophage cells. West blotting showed that briaranes **2**, **3**, and **6** inhibited LPS-induced iNOS expressions and briaranes **3** and **7** downregulated the expression of COX-2. Data were normalized to the cells treated with LPS only, and cells treated with dexamethasone (10 μM) were used as a positive control (which reduced the iNOS and COX-2 levels to 30.83 and 9.32%, respectively). Data are expressed as the mean ± SEM (*n* = 4). * Significantly different from cells treated with LPS (*p* < 0.05).

Using an in vitro cell culture model with a murine macrophage RAW264.7 cell line, anti-inflammatory analysis was performed to assess the activities of the compounds. Western blotting was used to measure the changes in pro-inflammatory proteins iNOS and COX-2 in the lipopolysaccharide (LPS)-induced pro-inflammatory response of RAW264.7 macrophages. In comparison with cells treated with LPS alone, the macrophages treated with a concentration of 10 μM, briaranes **2**, **3**, and **6** resulted in decreases in iNOS to 49.13, 36.22, and 43.33%, respectively, and **3** and **7** elicited reduction of COX-2 to 43.64 and 47.49%, respectively (Table 3 and Figure 4). Briaranes **1**–**7** did not cause significant cell death according to trypan blue staining, which suggested that the compounds had a low cytotoxicity towards the macrophage cells. Briarane **3** showed suppression effects on the expression of pro-inflammatory iNOS and COX-2 proteins, while **1** and **4** were observed to be inactive in terms of reducing the levels of these two proteins. These results suggested that the bulky acetate at C-12 could significantly enhance anti-inflammatory activities. 

## 3. Experimental Section

### 3.1. General Experimental Procedures

General experiment methods are as described in our previous study [23]. 

### 3.2. Animal Material

Specimens of the gorgonian coral *J. fragilis* were collected in June 2017 by hand by scuba divers off the coast of Southern Taiwan. The samples were then stored in a freezer until extraction. A voucher specimen was deposited in the National Museum of Marine Biology and Aquarium, Taiwan (NMMBA-TW-GC-2017-017). Identification of the species of this organism was performed by comparison as described in previous publication [1,2,3]. 

### 3.3. Extraction and Isolation

Sliced bodies of *J. fragilis* (wet weight 929 g, dry weight 374 g) were extracted with a mixture of methanol (MeOH) and dichloromethane (CH_2_Cl_2_) (v:v = 1:1). The extract (20.3 g) was partitioned between ethyl acetate (EtOAc) and H_2_O. The EtOAc layer (8.7 g) was separated on silica gel and eluted with *n*-hexane/EtOAc/MeOH (stepwise, v:v:v = 100:0:0 to 100% MeOH) to yield 14 subfractions A–N. Fraction I was purified by NP-HPLC using a mixture of *n*-hexane/acetone (v:v= 3:1 at a flow rate of 5.0 mL/min) to afford 15 subfractions I1–I15. Fraction I7 was purified by RP-HPLC using a mixture of acetonitrile/H_2_O (v:v= 1:1 at a flow rate of 1.0 mL/min) to yield **4** (0.6 mg). Fraction I9 was purified by NP-HPLC using a mixture of CH_2_Cl_2_/acetone (v:v = 15:1 at a flow rate of 2.0 mL/min) to yield **3** (81.9 mg) and **6** (0.7 mg). Fraction I14 was purified by NP-HPLC using a mixture of CH_2_Cl_2_/acetone (v:v = 15:1 at a flow rate of 2.0 mL/min) to yield **5** (0.9 mg) and **7** (2.7 mg). Fraction J was separated by silica gel column chromatography and then eluted with CH_2_Cl_2_/acetone (stepwise, v:v = 20:1 to 100% acetone) to afford 15 subfractions J1–J15. Fraction J12 was purified by NP-HPLC using a mixture of *n*-hexane/acetone (v:v = 2:1 at a flow rate of 2.0 mL/min) to yield **2** (1.2 mg). Fraction L was separated by silica gel column chromatography and then eluted with *n*-hexane/acetone (stepwise, v:v = 50:1 to 100% acetone) to afford eleven subfractions L1–L11. Fraction L9 was separated by silica gel column chromatography and then eluted with CH_2_Cl_2_/acetone (stepwise, v:v = 80:1 to 100% acetone) to afford 11 subfractions L9A–L9K. Fraction L9I was purified by NP-HPLC using a mixture of CH_2_Cl_2_/acetone (v:v = 15:1) to afford seven subfractions L9I1–L9I7. Fraction L9I2 was purified by RP-HPLC using a mixture of MeOH/H_2_O (v:v = 80:20 at a flow rate of 2.0 mL/min) to yield **1** (0.8 mg). 

Fragilide K (**1**): amorphous powder; mp 136–138 °C; [α]${}_{D}^{22}$ −43 (*c* 0.02, CHCl_3_); IR (neat) ν_max_ 3545, 1790, 1740 cm^−1^; ^1^H (400 MHz, CDCl_3_) and ^13^C (100 MHz, CDCl_3_) NMR data (see Table 1); ESIMS: *m*/*z* 637 [M + Na]^+^, 639 [M + 2 + Na]^+^; HRESIMS: *m*/*z* 637.16613 (calcd. for C_28_H_35_ClO_13_ + Na, 637.16584).

Fragilide L (**2**): amorphous powder; mp 129–131 °C; [α]${}_{D}^{22}$ −75 (*c* 0.06, CHCl_3_); IR (neat) ν_max_ 3447, 1771, 1731 cm^−1^; ^1^H (400 MHz, CDCl_3_) and ^13^C (100 MHz, CDCl_3_) NMR data (see Table 2); ESIMS: *m*/*z* 531 [M + Na]; HRESIMS: *m*/*z* 531.22014 (calcd. for C_26_H_36_O_10_ + Na, 531.22007).

### 3.4. Molecular Mechanics Calculations

Implementation of the MM2 force field [19] program in ChemBio 3D Ultra software from Cambridge Soft Corporation (ver. 12.0, Cambridge, MA, USA) was used to create molecular models. 

### 3.5. In Vitro Anti-Inflammatory Assay

Murine macrophage-like cell line RAW264.7 was purchased from the American Type Culture Collection (ATCC, No TIB-71) (Manassas, VA, USA). The in vitro anti-inflammatory activities of compounds **1**–**7** were assessed by investigating their inhibition effects on LPS-induced pro-inflammatory iNOS and COX-2 protein expressions in the macrophage cell line using western blot analysis [24,25,26]. Briefly, inflammation in macrophages was induced by incubating them for 16 h in a medium containing only LPS (10 μM) without compounds. For the anti-inflammatory activity assays, Briaranes **1**–**7** and dexamethasone (10 μM) were added to the cells 10 min before LPS challenge. The cells were then subjected to western blot analysis. The immunoreactivity data were calculated with respect to the average optical density of the corresponding LPS-stimulated group. The RAW264.7 macrophage cell viability was determined after treatment with alamar blue (invitrogen, Carlsbad, CA, USA), a tetrazolium dye that is reduced by living cells to fluorescent products. This assay is similar in principle to the cell viability assay using 3-(4,5-dimethldiazol-2-yl)-2,5-diphenyltetrazolium bromide and has been validated as an accurate measure of the survival of RAW264.7 macrophage cells [27,28]. For statistical analysis, the data was analyzed by one-way analysis of variance (ANOVA), followed by the Student–Newman–Keuls *post hoc* test for multiple comparison. A significant difference was defined as a *p*-value of < 0.05. 

## 4. Conclusions

Gorgonian corals belonging to the family Ellisellidae have proven to be a rich source of interesting polyoxygenated and chlorinated briarane-related natural products with complex structures and extensive bioactivities. Fragilide L (**2**), gemmacolides X (**3**) and V (**7**), and juncin ZI (**6**), are compounds suitable for further study, as they have the potential to be developed as new medicinal agents. Among these compounds, we suggested that gemmacolide X (**3**) has more potential for its mass production from the target pharmaceutical-origin material *J. fragilis*. This interesting coral species has been transferred to culture tanks located in our institute to produce a large quantity of culture material. The cultured *J. fragilis* is used for the isolation of natural raw ingredients in order to establish a constant supply of bioactive materials.

## Figures and Tables

**Table 1:** ^1^H (400 MHz, CDCl_3_) and ^13^C (100 MHz, CDCl_3_) NMR data, ^1^H–^1^H COSY, and HMBC correlations for briarane **1**.

C/H	δ_H_ (*J* in Hz)	δ_C_, Multiple	^1^H–^1^H COSY	HMBC (H→C)
1		46.9, C		
2	5.50 d (6.8)	72.6, CH	H-3	C-1, C-3, C-4, C-15, acetate carbonyl
3	6.22 dd (10.8, 6.8)	63.7, CH	H-2, H-4	C-1, C-4, C-5, acetate carbonyl
4	4.49 d (10.8)	78.9, CH	H-3	C-3, C-5, C-6, C-8, C-16
5		134.1, C		
6	4.96 d (2.8)	53.8, CH	H-7	n. o.
7	4.42 d (2.8)	79.0, CH	H-6	C-5
8		83.0, C		
9	5.60 s	71.1, CH	n. o. ^a^	C-1, C-10, C-11, C-17, acetate carbonyl
10	3.32 s	35.1, CH	n. o.	C-1, C-8, C-11, C-12, C-15, C-20
11		58.9, C		
12	3.49 br s	72.2, CH	H_2_-13	n. o.
13α/β	2.20 ddd (15.6, 3.2, 2.8); 1.98 ddd (15.6, 3.2, 3.2)	30.3, CH_2_	H-12, H-14	n. o.
14	5.01 dd (3.2, 3.2)	74.2, CH	H_2_-13	n. o.
15	1.24 s	15.2, CH_3_		C-1, C-2, C-10, C-14
16a/b	5.36 d (1.6); 5.57 d (1.6)	119.7, CH_2_		C-4, C-6
17	2.79 q (7.2)	49.6, CH	H_3_-18	C-18, C-19
18	1.37 d (7.2)	7.4, CH_3_	H-17	C-8, C-17, C-19
19		174.2, C		
20a/b	2.76 d (3.2); 2.56 d (3.2)	50.6, CH_2_		n. o.
OAc-2		170.2, C		
	2.05 s	20.5, CH_3_		Acetate carbonyl
OAc-3		169.9, C		
	2.08 s	21.1, CH_3_		Acetate carbonyl
OAc-9		169.5, C		
	2.32 s	21.1, CH_3_		Acetate carbonyl
OAc-14		169.9, C		
	2.00 s	20.3, CH_3_		Acetate carbonyl

^a^ n. o. = not observed.

**Table 2:** ^1^H (400 MHz, CDCl_3_) and ^13^C (100 MHz, CDCl_3_) NMR data, ^1^H–^1^H COSY and HMBC correlations for briarane **2**

C/H	δ_H_ (*J* in Hz)	δ_C_, Multiple	^1^H–^1^HCOSY	HMBC (H→C)
1		47.0, C		
2	4.83 dd (6.0, 1.2)	75.9, CH	H_2_-3	C-1, C-3, C-4, C-10, C-15, acetate carbonyl
3α/β	1.72 m; 2.49 m	31.8, CH_2_	H-2, H_2_-4	C-1, C-4
4α/β	2.26 m; 2.59 m	26.2, CH_2_	H_2_-3	C-3, C-5, C-6, C-16
5		146.7, C		
6	5.94 d (10.0)	121.7, CH	H-7	C-4, C-16
7	5.30 d (10.0)	77.3, CH	H-6	C-5, C-6, C-8
8		83.2, C		
9	5.30 d (5.2)	71.5, CH	H-10	C-1, C-7, C-8, C-10, C-11, acetate carbonyl
10	3.31 d (5.2)	42.2, CH	H-9	C-1, C-2, C-8, C-9, C-11, C-12, C-15, C-20
11		150.9, C		
12a/b	2.26 m; 2.18 m	26.4, CH_2_	H_2_-13	C-11, C-13, C-14, C-20
13a/b	2.01 m; 1.76 m	27.4, CH_2_	H_2_-12, H-14	C-1, C-11, C-14
14	4.72 dd (4.8, 1.6)	73.8, CH	H_2_-13	C-1, C-2, C-10, C-12, C-13, C-15,
				acetate carbonyl
15	1.09 s	15.1, CH_3_		C-1, C-2, C-10, C-14
16a/b	4.28 dd (14.0, 5.2); 4.14 dd (14.0, 7.6)	68.9, CH_2_	OH-16	C-5, C-6
17	2.47 q (7.2)	42.6, CH	H_3_-18	C-8, C-18, C-19
18	1.13 d (7.2)	6.6, CH_3_	H-17	C-8, C-17, C-19
19		175.8, C		
20a/b	4.90 s; 5.06 s	113.1, CH_2_		C-10, C-11, C-12
OAc-2		171.8, C		
	2.01 s	21.1, CH_3_		Acetate carbonyl
OAc-9		169.4, C		
	2.21 s	21.8, CH_3_		Acetate carbonyl
OAc-14		170.6, C		
	1.91 s	21.2, CH_3_		Acetate carbonyl
OH-8	2.05 s			C-7, C-8, C-9, C-17
OH-16	3.01 dd (7.6, 5.2)		H_2_-16	n. o. ^a^

^a^ n. o. = not observed.

**Table 3:** Effects of **1**–**7** on iNOS and COX-2 protein expressions in LPS-stimulated macrophages.

Compound	iNOS	COX-2	β-Actin
	Expression (% of LPS Group)	Expression (% of LPS Group)	Expression (% of LPS Group)
Control	1.01 ± 0.15	7.07 ± 3.88	86.12 ± 8.75
LPS	100 ± 11.26	100 ± 3.36	100 ± 0.07
**1**	61.22 ± 3.82	88.09 ± 6.87	94.26 ± 2.3
**2**	49.13 ± 4.15	80.08 ± 7.98	110.11 ± 3.16
**3**	36.22 ± 13.28	43.64 ± 6.23	99.30 ± 16.53
**4**	61.63 ± 21.36	81.55 ± 22.66	117.99 ± 6.04
**5**	76.16 ± 21.90	58.94 ± 0.8	117.48 ± 13.63
**6**	43.33 ± 10.82	68.87 ± 11.08	129.76 ± 25.75
**7**	74.65 ± 18.02	47.49 ± 1.49	124.60 ± 18.10
Dex. ^a^	30.83 ± 6.69	9.32 ± 3.47	100.88 ± 3.14

^a^ Dexamethasone (Dex., 10 μM) was used as a positive control.

## Supplementary Materials

HRESIMS, IR, 1D (^1^H NMR, ^13^C NMR, and DEPT spectra), and 2D (HSQC, ^1^H−^1^H COSY, HMBC, and NOESY spectra) NMR spectra of new compounds **1** and **2** are available online.
