# Supplementary material for: Fragilides K and L, New Briaranes from the Gorgonian Coral Junceella fragilis
Source: Molecules. 2018 Jun 22;23(7):1510. doi: 10.3390/molecules23071510 (PMC6100390; doi:10.3390/molecules23071510)
Supplement: Supplementary file 1 [file molecules-23-01510-s001.pdf]

Supporting Information

Fragilides K and L, New Briaranes from the Gorgonian Coral *Junceella fragilis*

No	Content	page
S1.	HRESIMS spectrum of compound **1**.	2
S2.	IR spectrum of compound **1**.	2
S3.	^{1}H NMR spectrum (400 MHz) of compound **1** in $CDCl_3$.	3
S4.	^{13}C NMR spectrum (100 MHz) of compound **1** in $CDCl_3$.	3
S5.	DEPT spectrum of compound **1** in $CDCl_3$.	4
S6.	gHSQC spectrum of compound **1** in $CDCl_3$.	4
S7.	^{1}H–^{1}H gCOSY spectrum of compound **2** in $CDCl_3$.	5
S8.	gHMBC spectrum of compound **1** in $CDCl_3$.	5
S9.	NOESY spectrum of compound **1** in $CDCl_3$.	6
S10.	HRESIMS spectrum of compound **2**.	6
S11.	IR spectrum of compound **2**.	7
S12.	^{1}H NMR spectrum (400 MHz) of compound **2** in $CDCl_3$.	7
S13.	^{13}C NMR spectrum (100 MHz) of compound **2** in $CDCl_3$.	8
S14.	DEPT spectrum of compound **2** in $CDCl_3$.	8
S15.	gHSQC spectrum of compound **2** in $CDCl_3$.	9
S16.	^{1}H–^{1}H gCOSY spectrum of compound **2** in $CDCl_3$.	9
S17.	gHMBC spectrum of compound **2** in $CDCl_3$.	10
S18.	NOESY spectrum of compound **2** in $CDCl_3$.	10

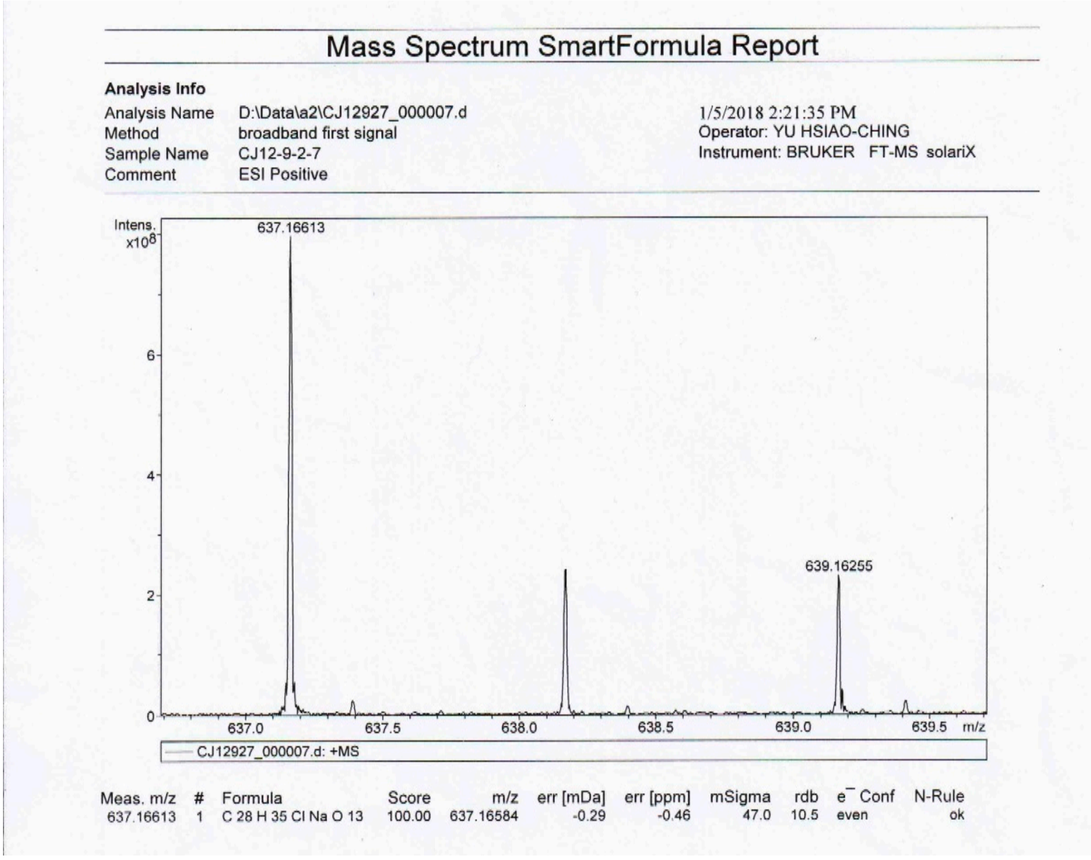

S1. HRESIMS spectrum of compound **1**.

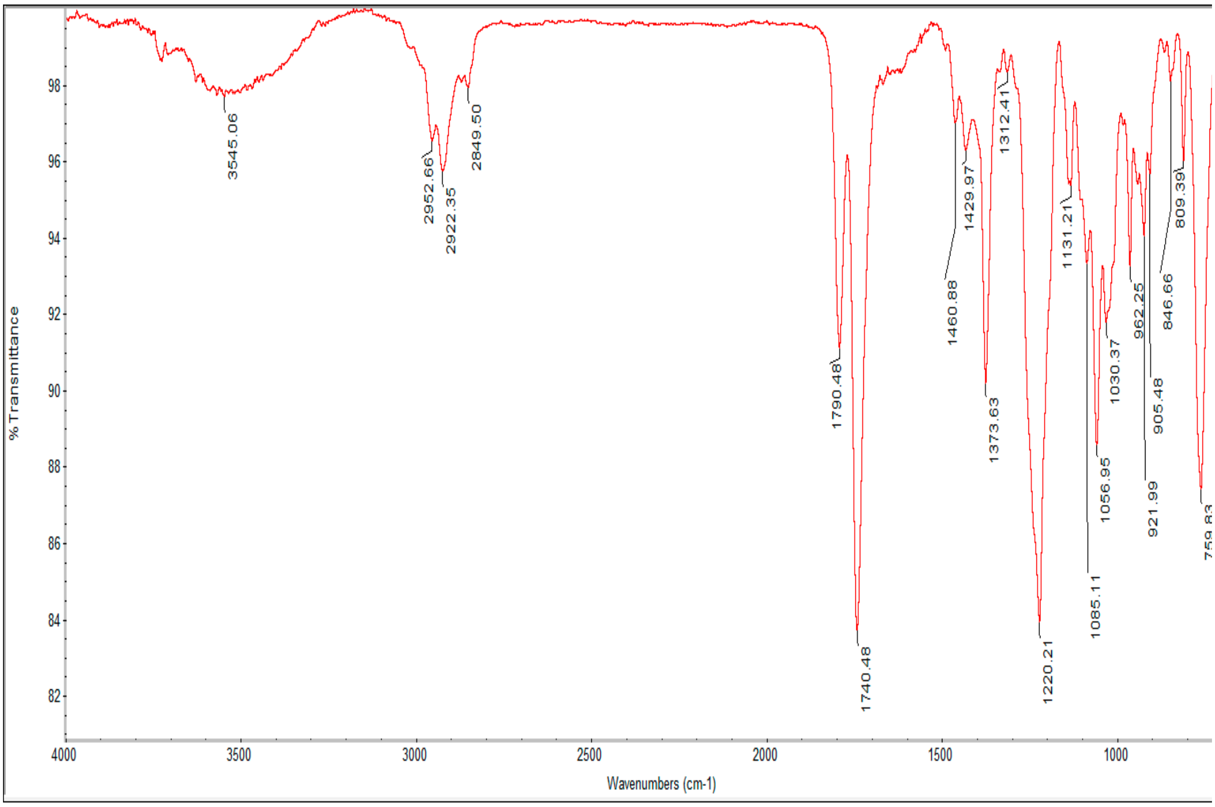

S2. IR spectrum of compound **1**.

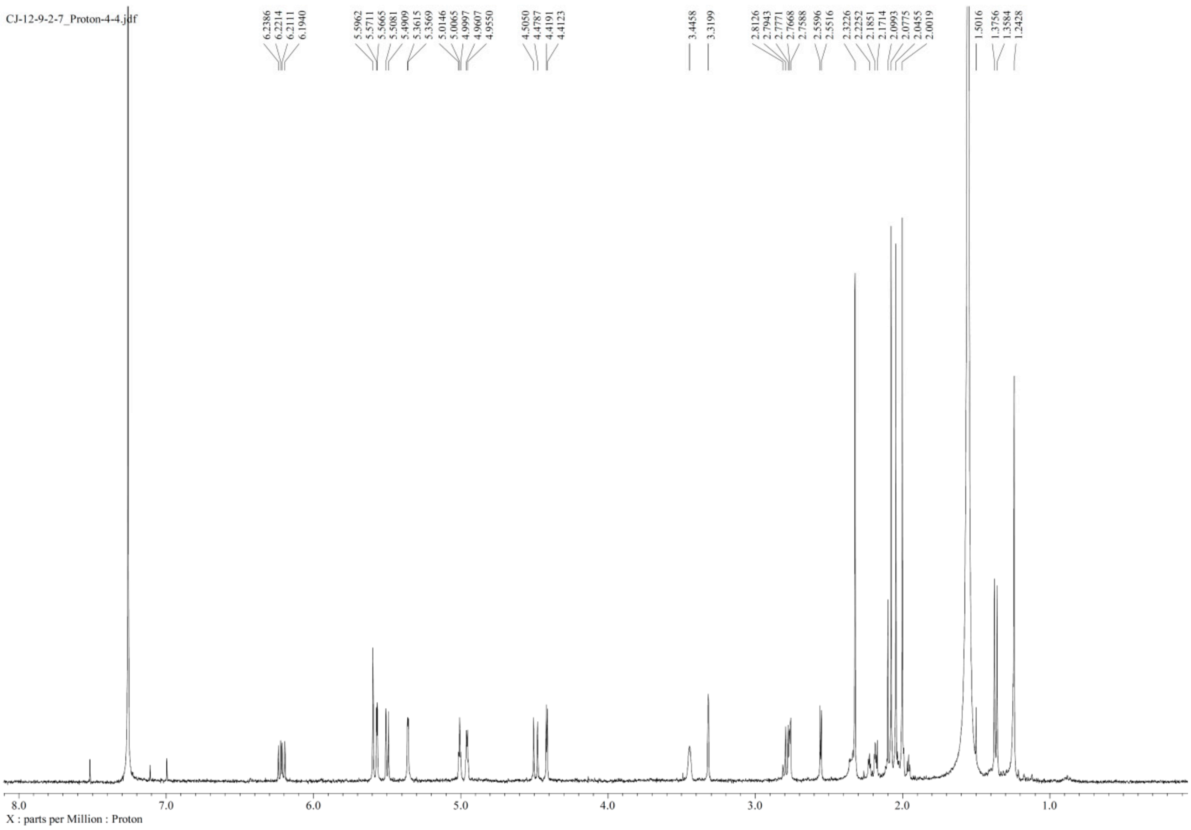

S3. ^{1}H NMR spectrum (400 MHz) of compound **1** in $CDCl_3$

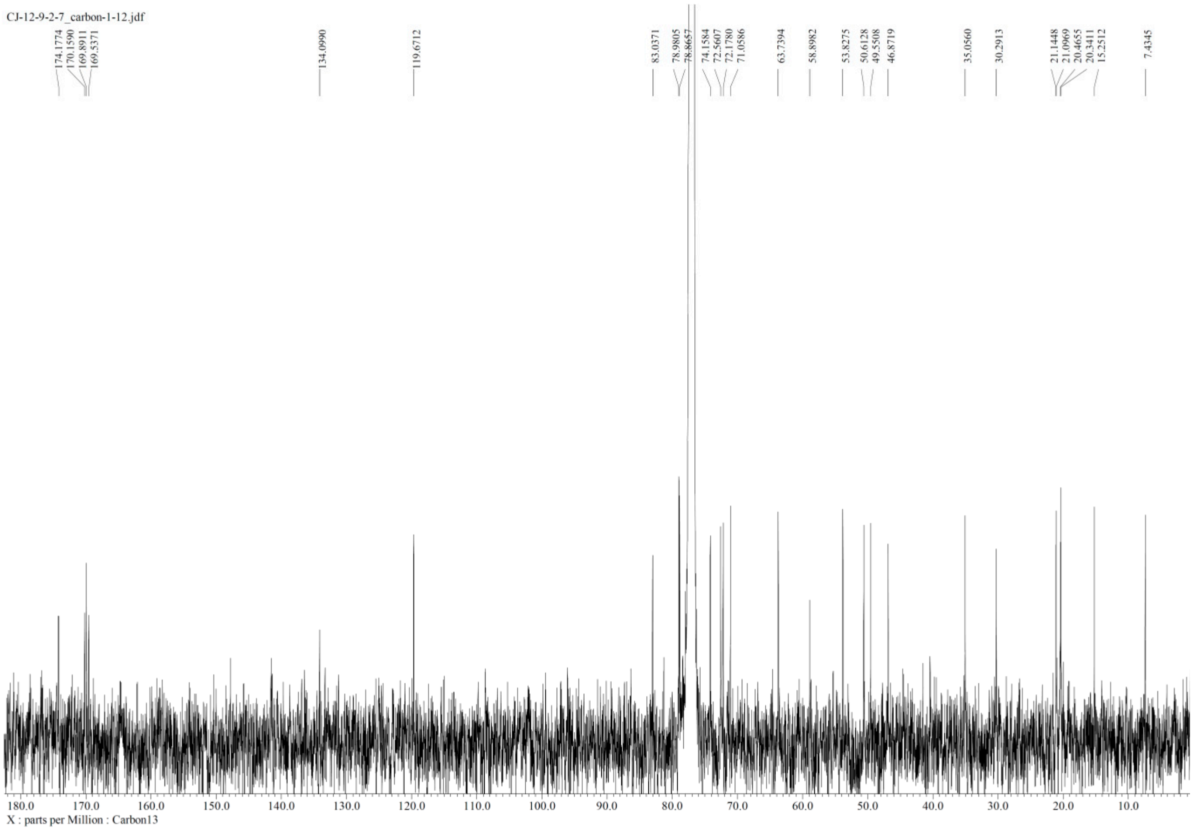

S4. 13C NMR spectrum (100 MHz) of compound 1 in CDCl3.

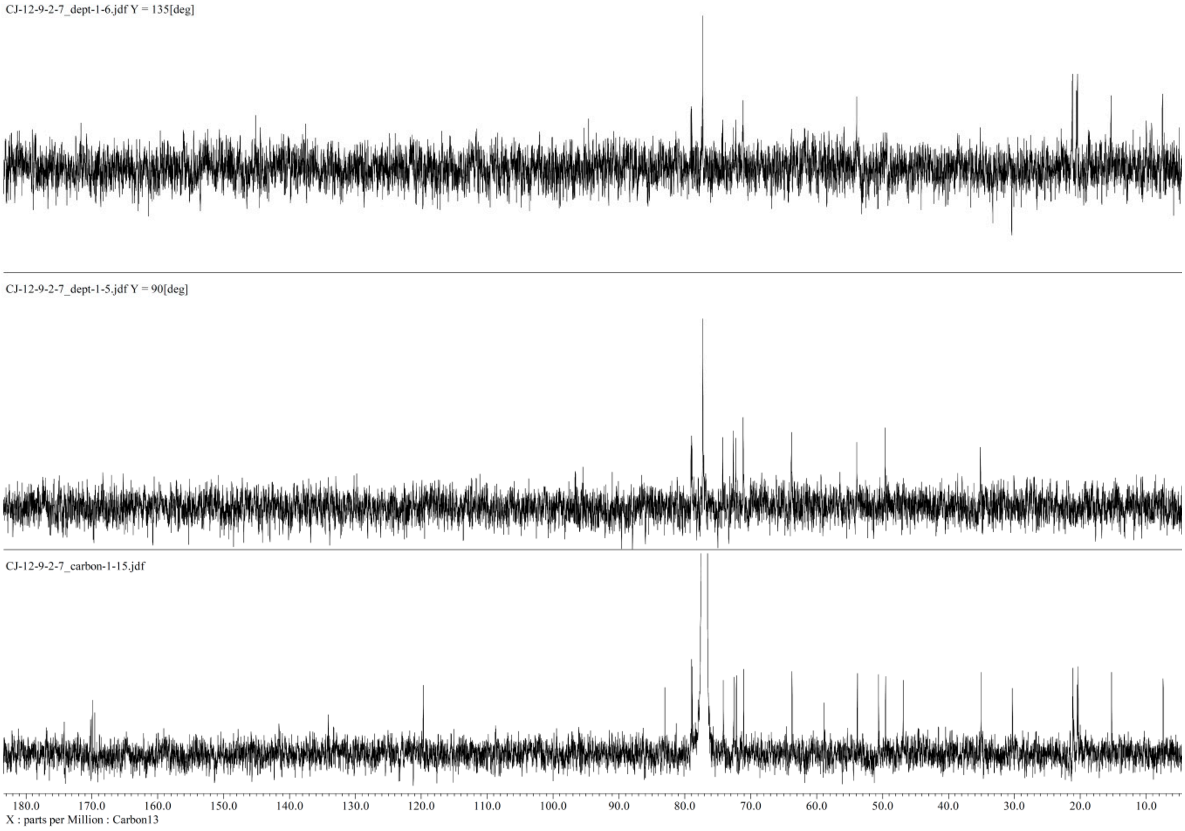

S5. DEPT spectrum of compound **1** in $CDCl_3$.

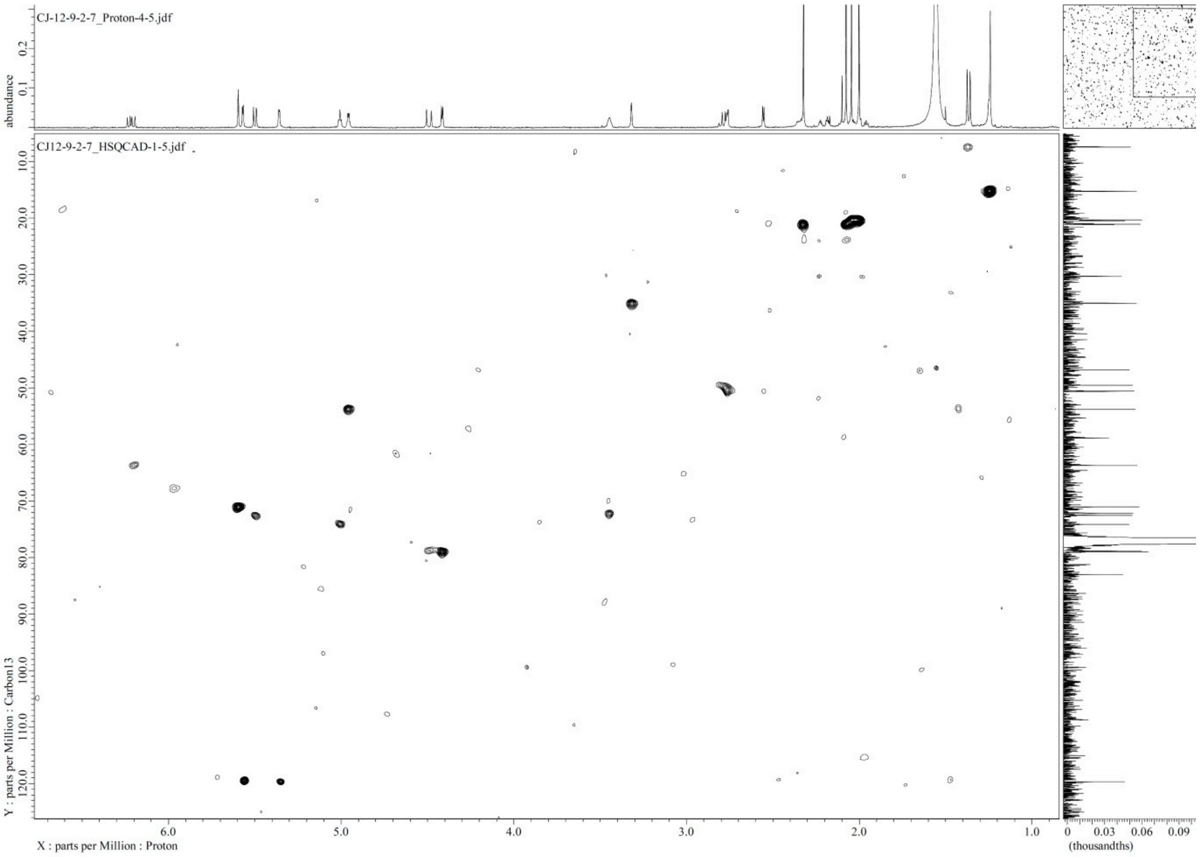

S6. gHSQC spectrum of compound **1** in $CDCl_3$.

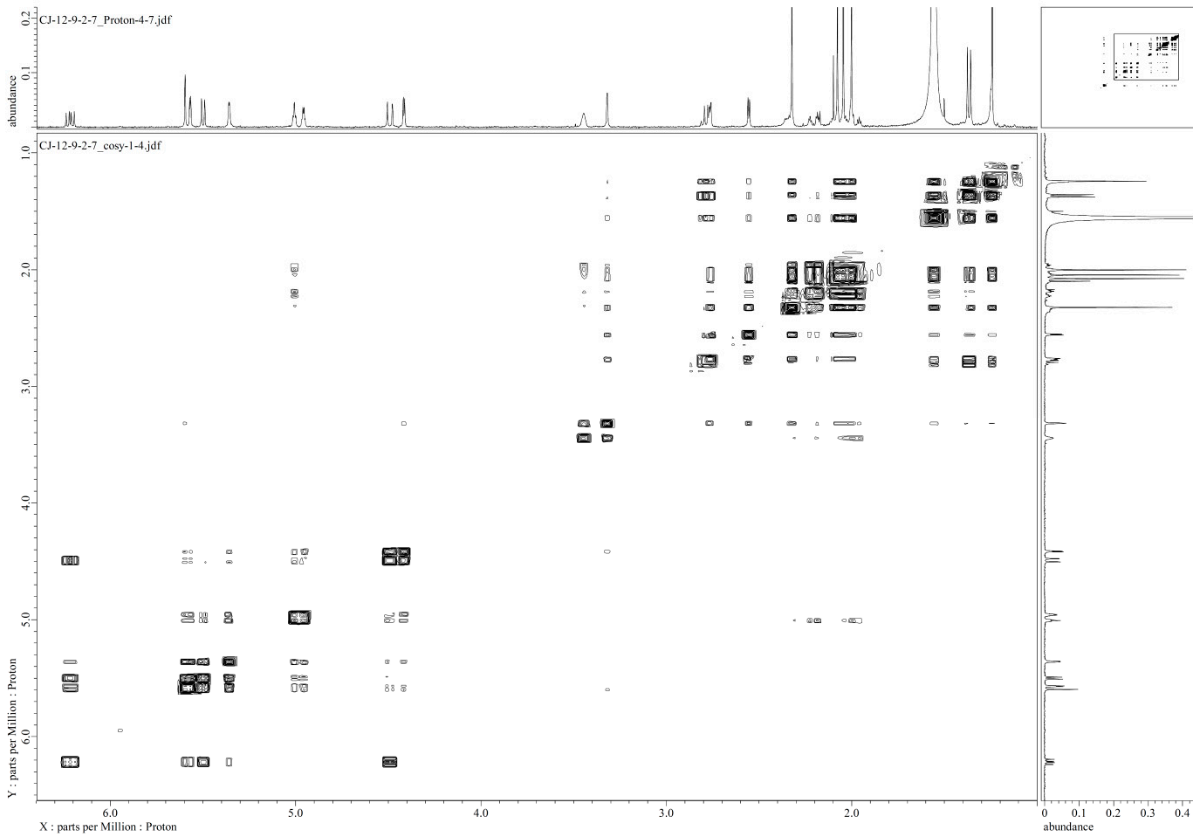

S7. ^{1}H–^{1}H gCOSY spectrum of compound **1** in CDCl$_3$.

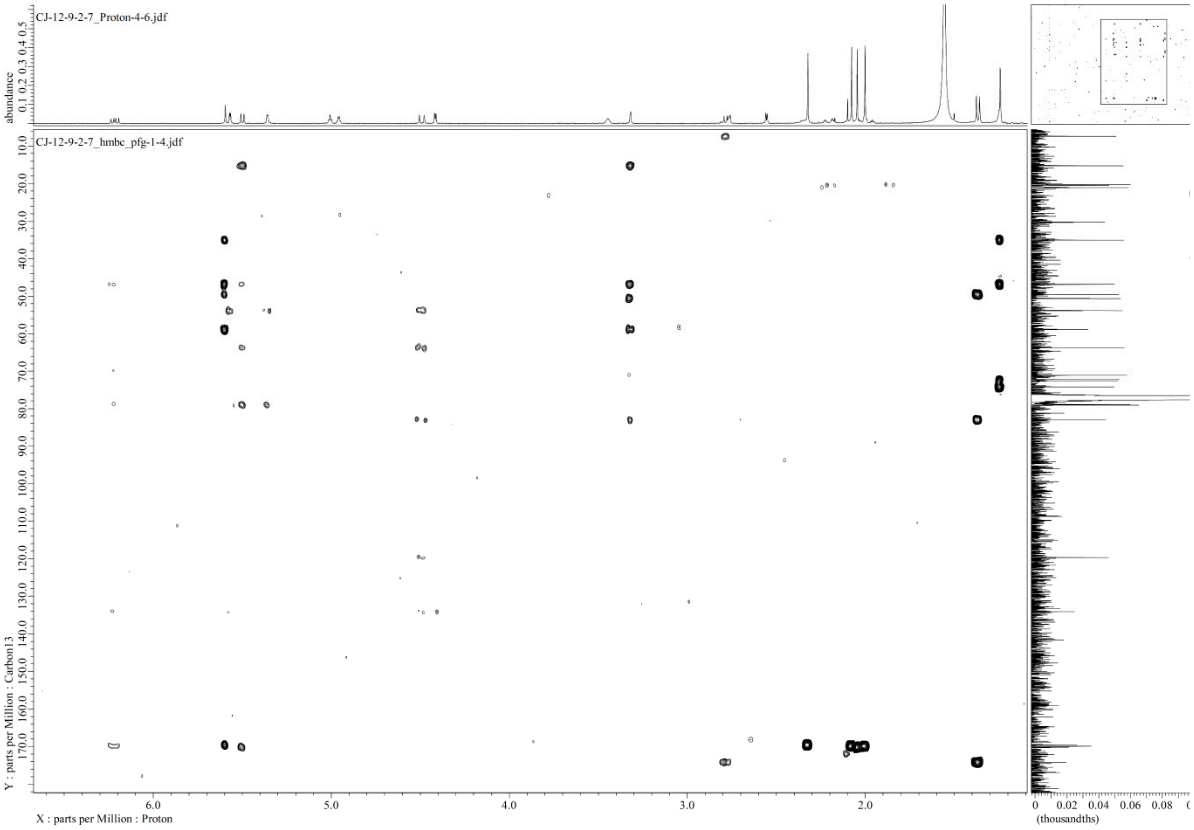

S8. gHMBC spectrum of compound **1** in $CDCl_3$.

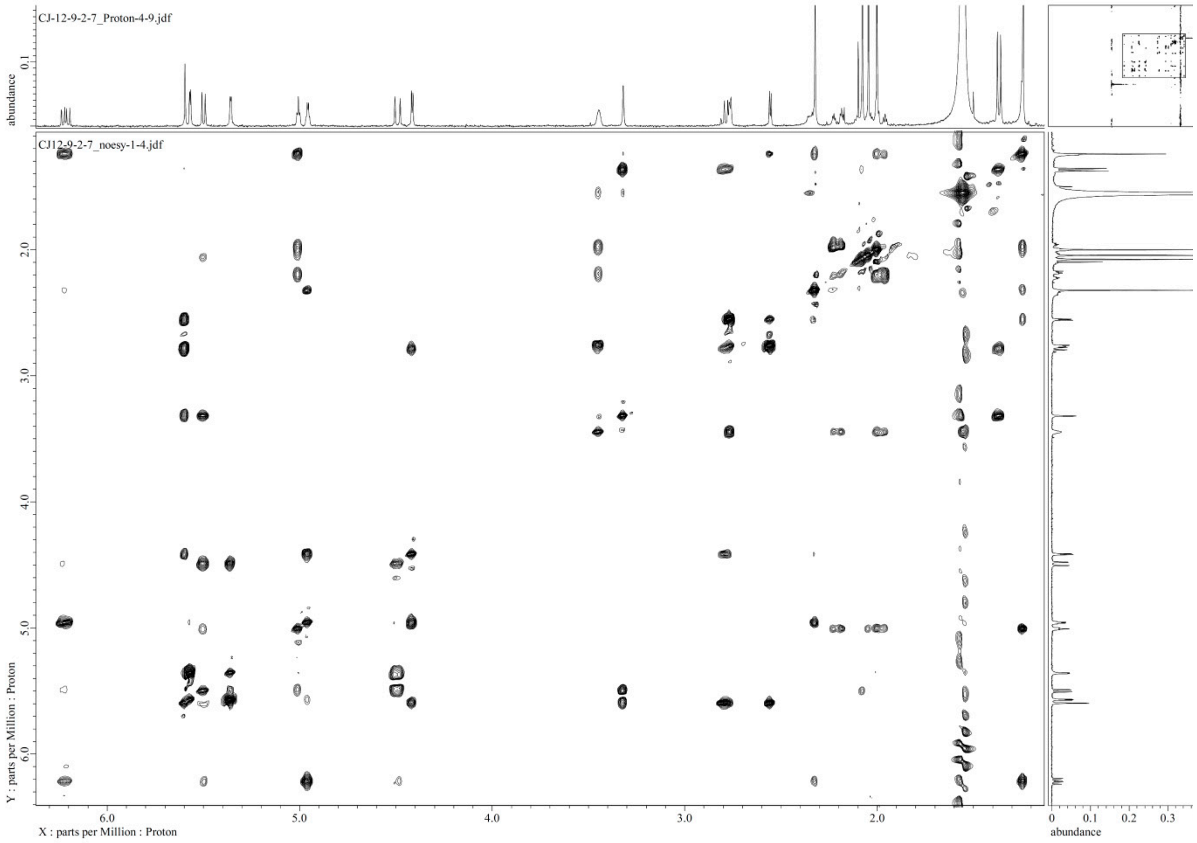

S9. NOESY spectrum of compound **1** in $CDCl_3$.

Mass Spectrum SmartFormula Report

Analysis Info

Analysis Name	D:\Data\a2\CJ10123_000002.d	2/6/2018 3:29:19 PM
Method	broadband first signal	Operator: YU HSIAO-CHING
Sample Name	CJ-10-12-3	Instrument: BRUKER FT-MS solariX
Comment	ESI Positive	

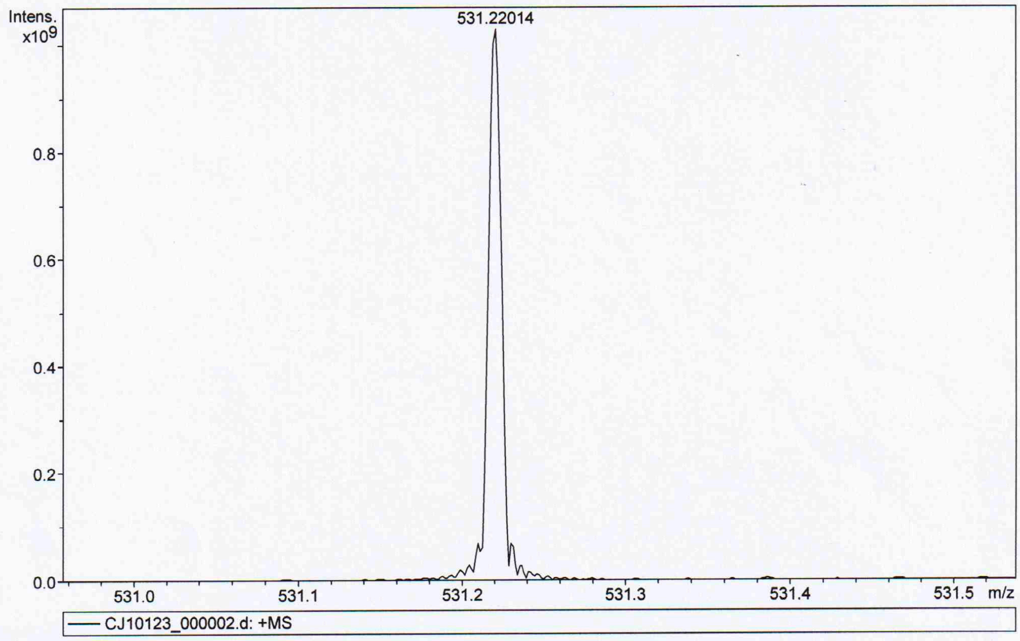

Meas. m/z	#	Formula	Score	m/z	err [mDa]	err [ppm]	mSigma	rdb	e⁻ Conf	N-Rule
531.22014	1	C 26 H 36 Na O 10	100.00	531.22007	-0.07	-0.13	9.9	8.5	even	ok

S10. HRESIMS spectrum of compound **2**.

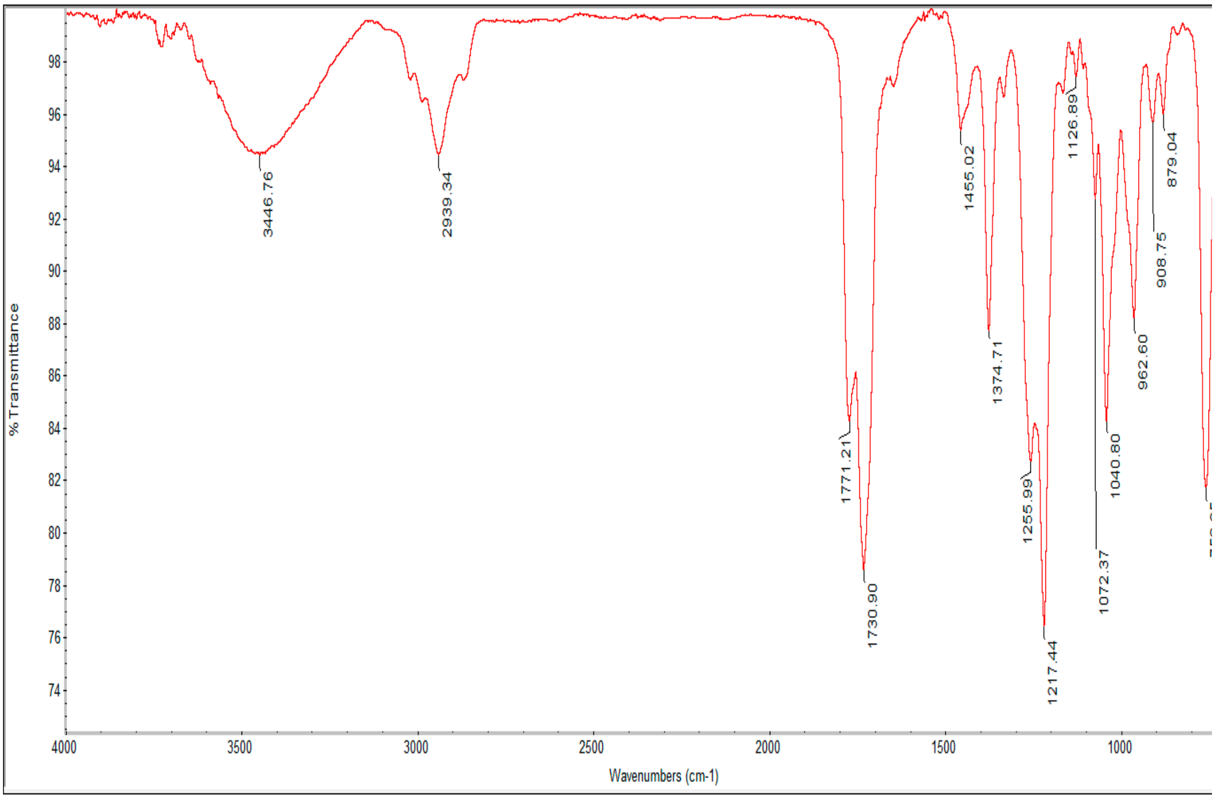

S11. IR spectrum of compound **2**.

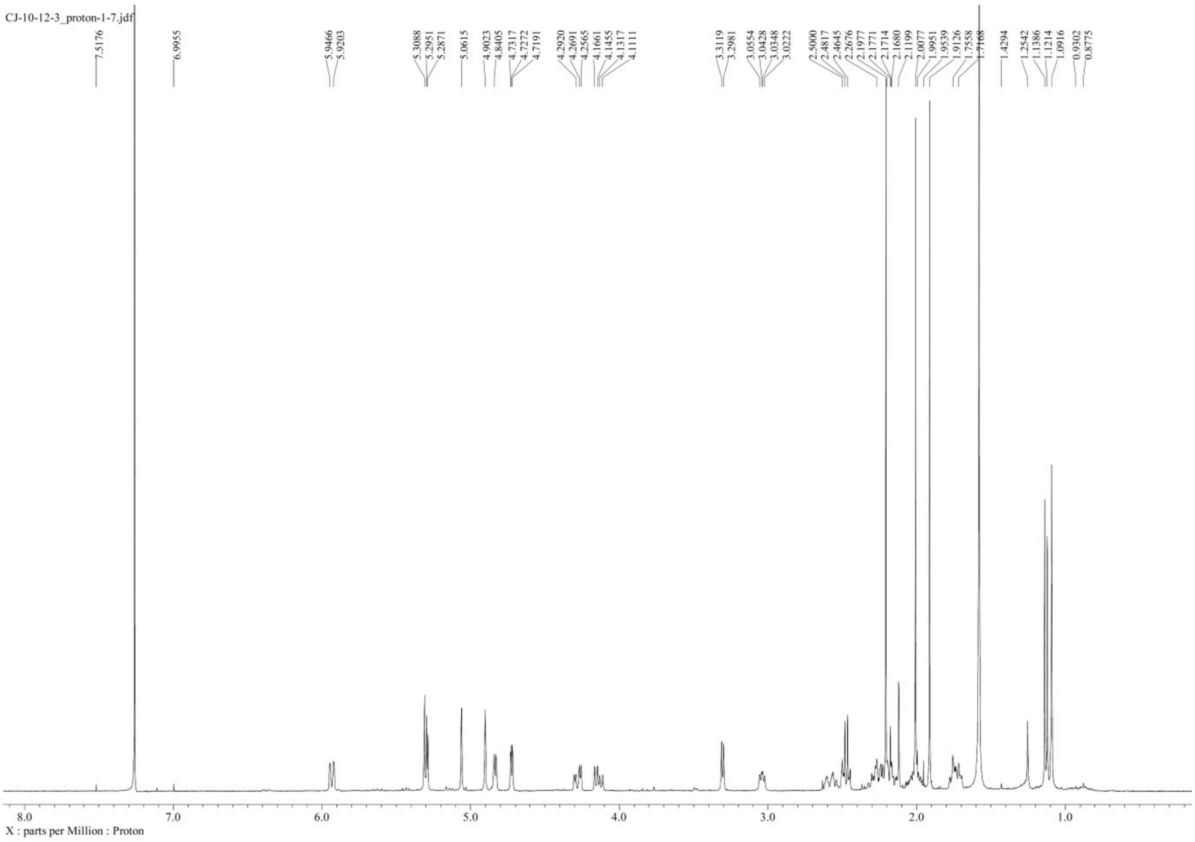

S12. ^{1}H NMR spectrum (400 MHz) of compound **2** in $CDCl_3$.

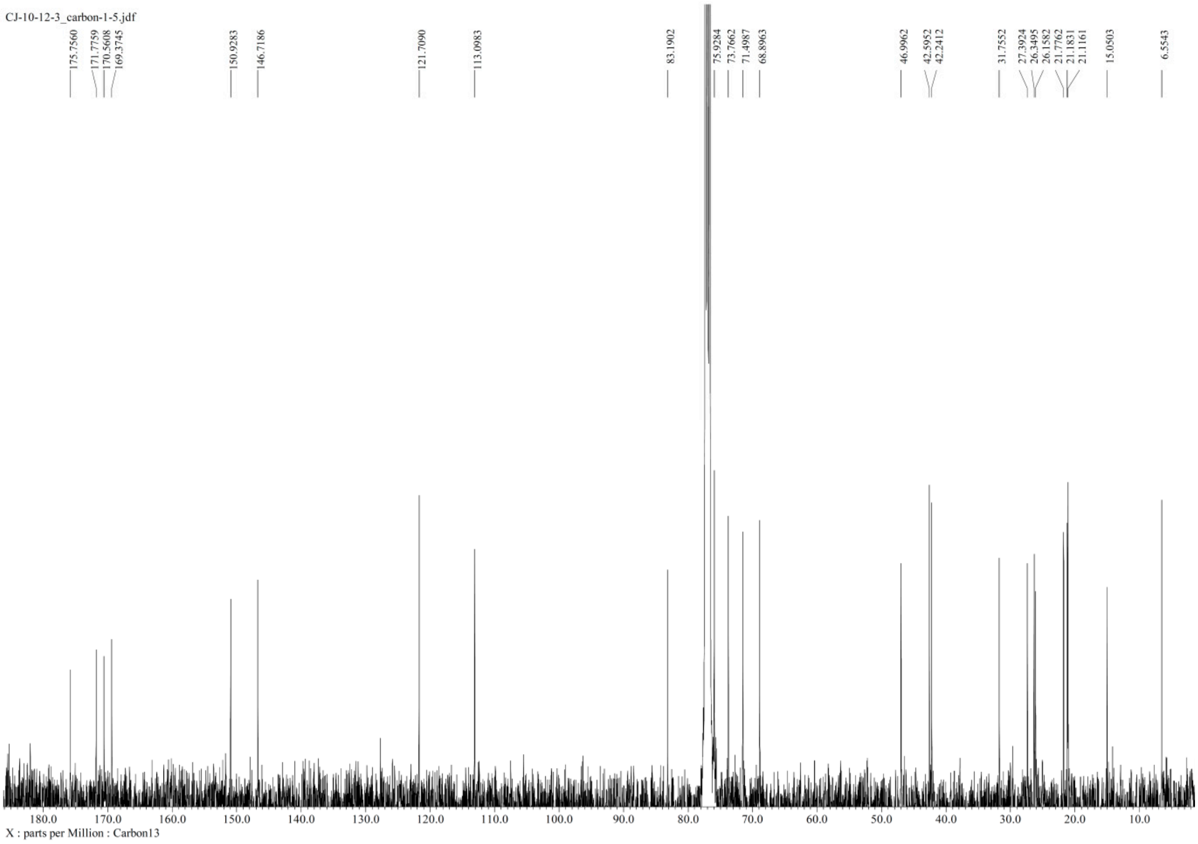

S13. ^{13}C NMR spectrum (100 MHz) of compound **2** in $CDCl_3$.

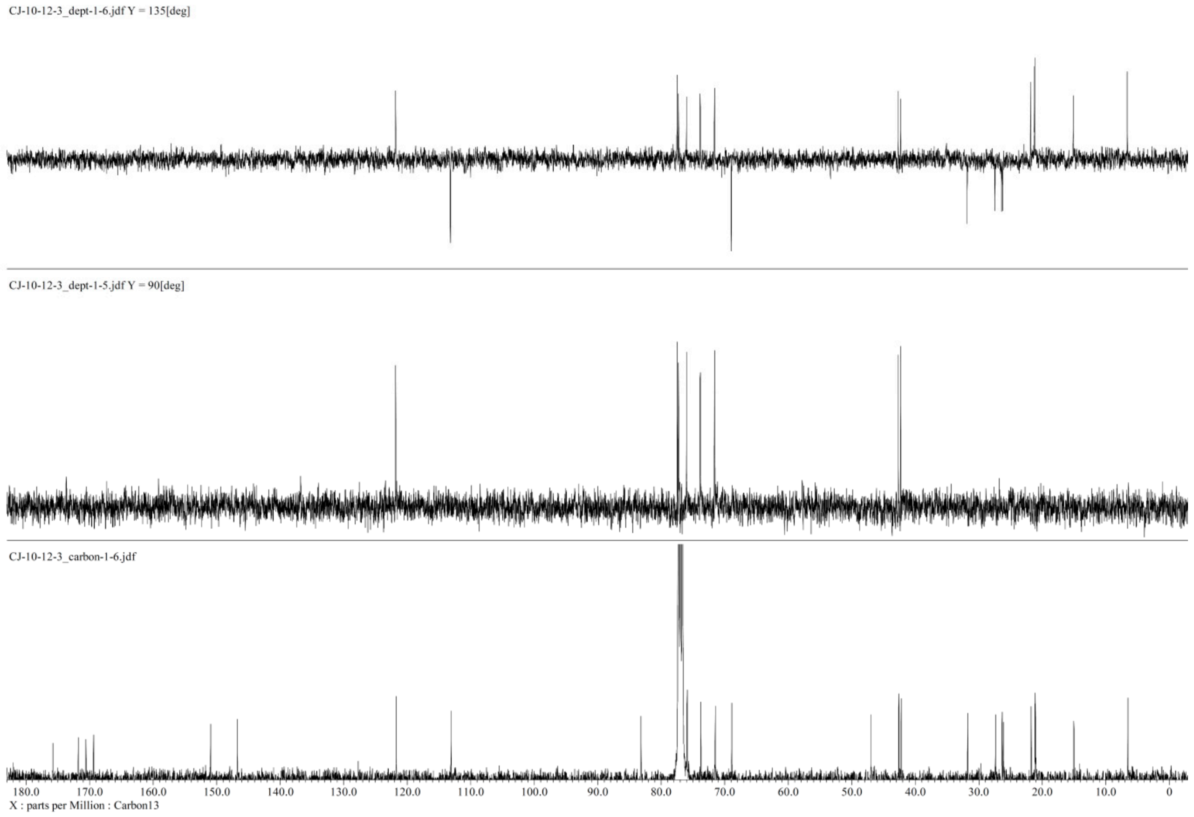

S14. DEPT spectrum of compound **2** in $CDCl_3$.

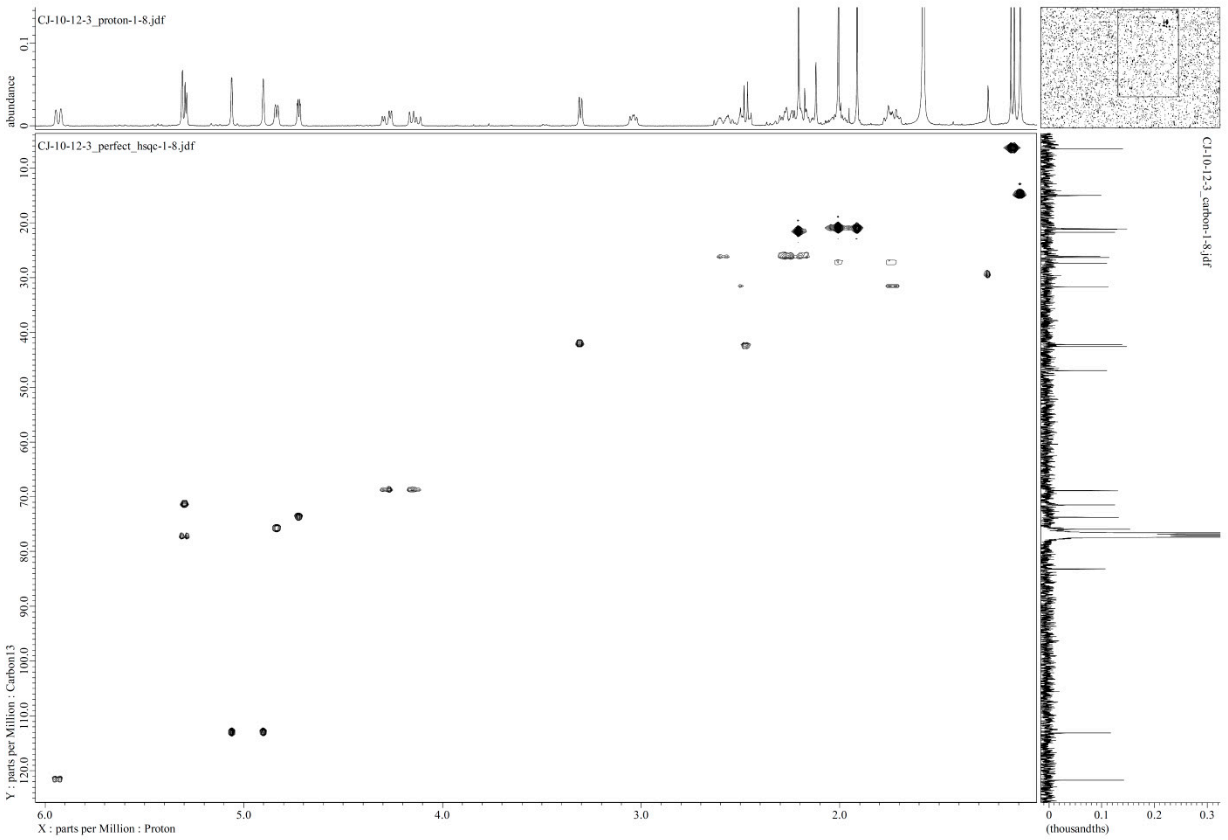

S15. gHSQC spectrum of compound **2** in $CDCl_3$.

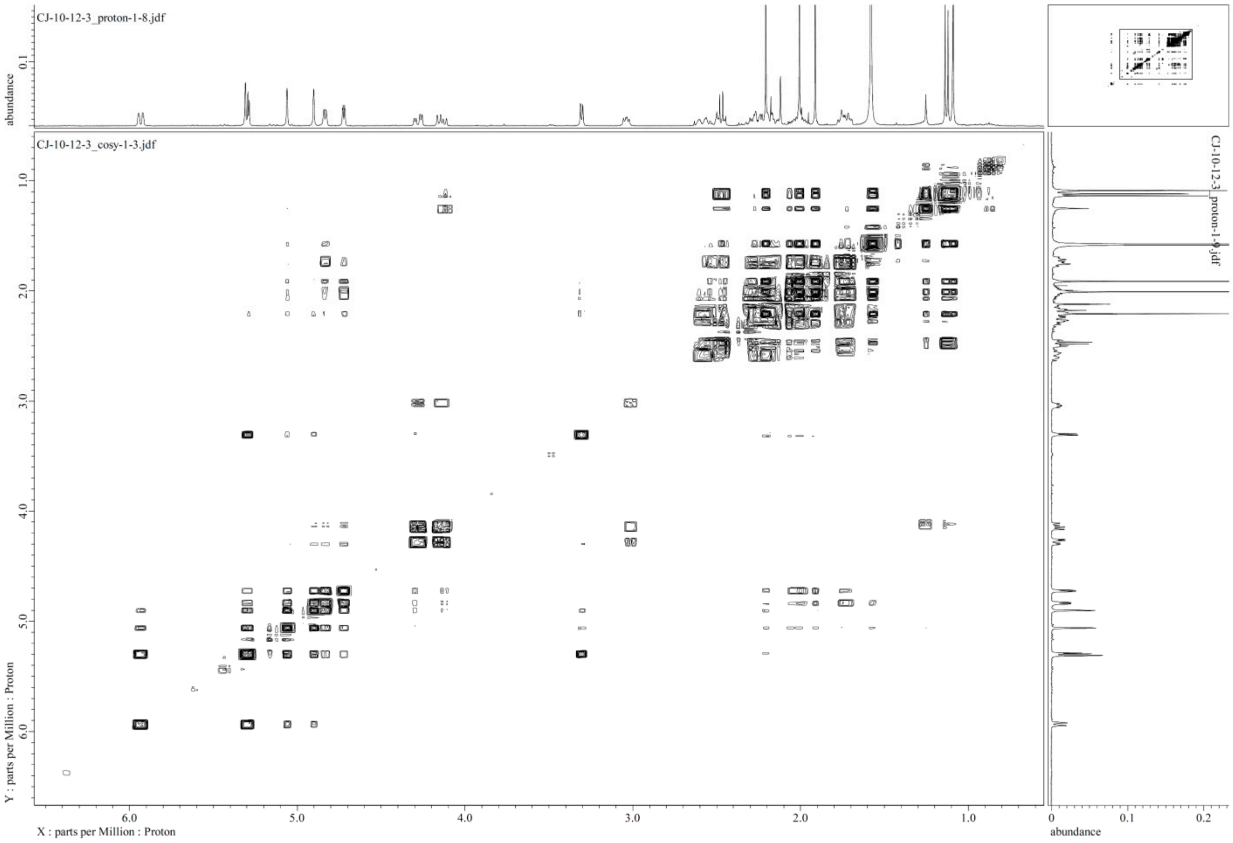

S16. ^{1}H–^{1}H gCOSY spectrum of compound **2** in $CDCl_3$.

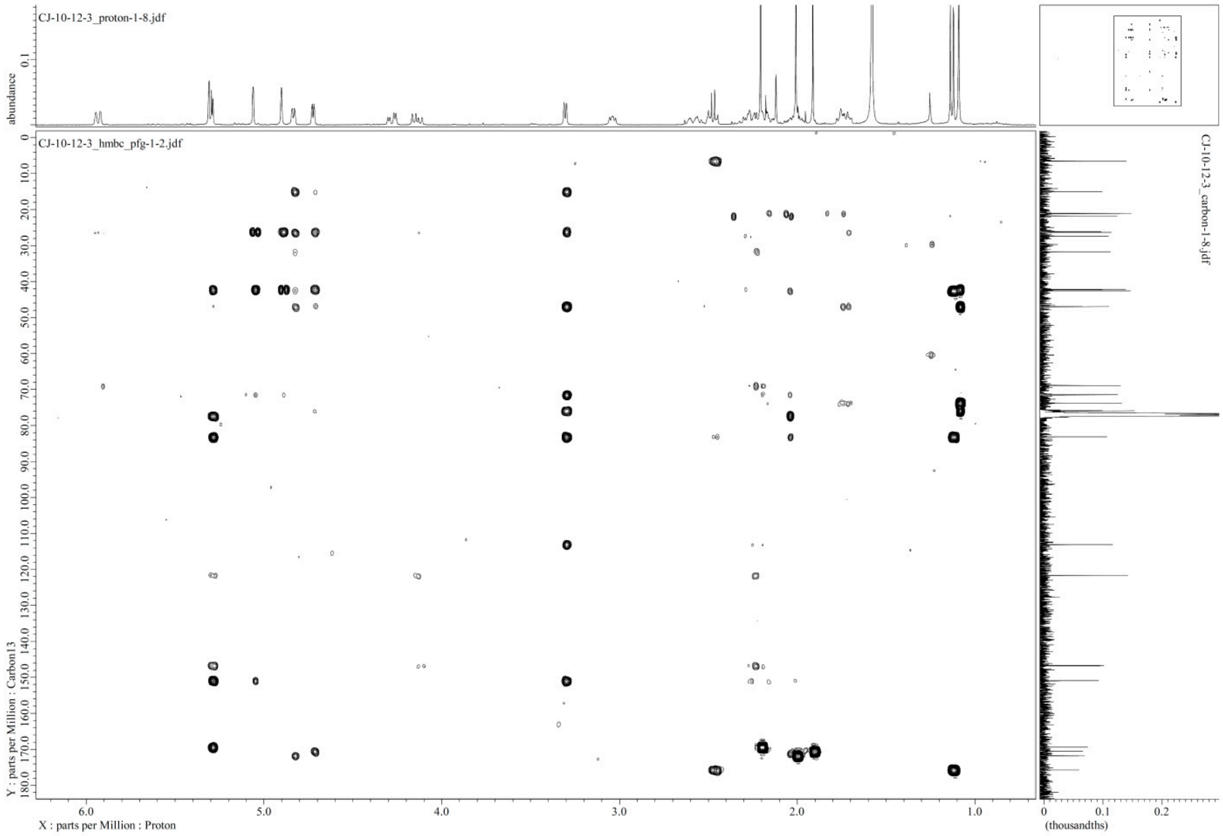

S17. gHMBC spectrum of compound **2** in $CDCl_3$.

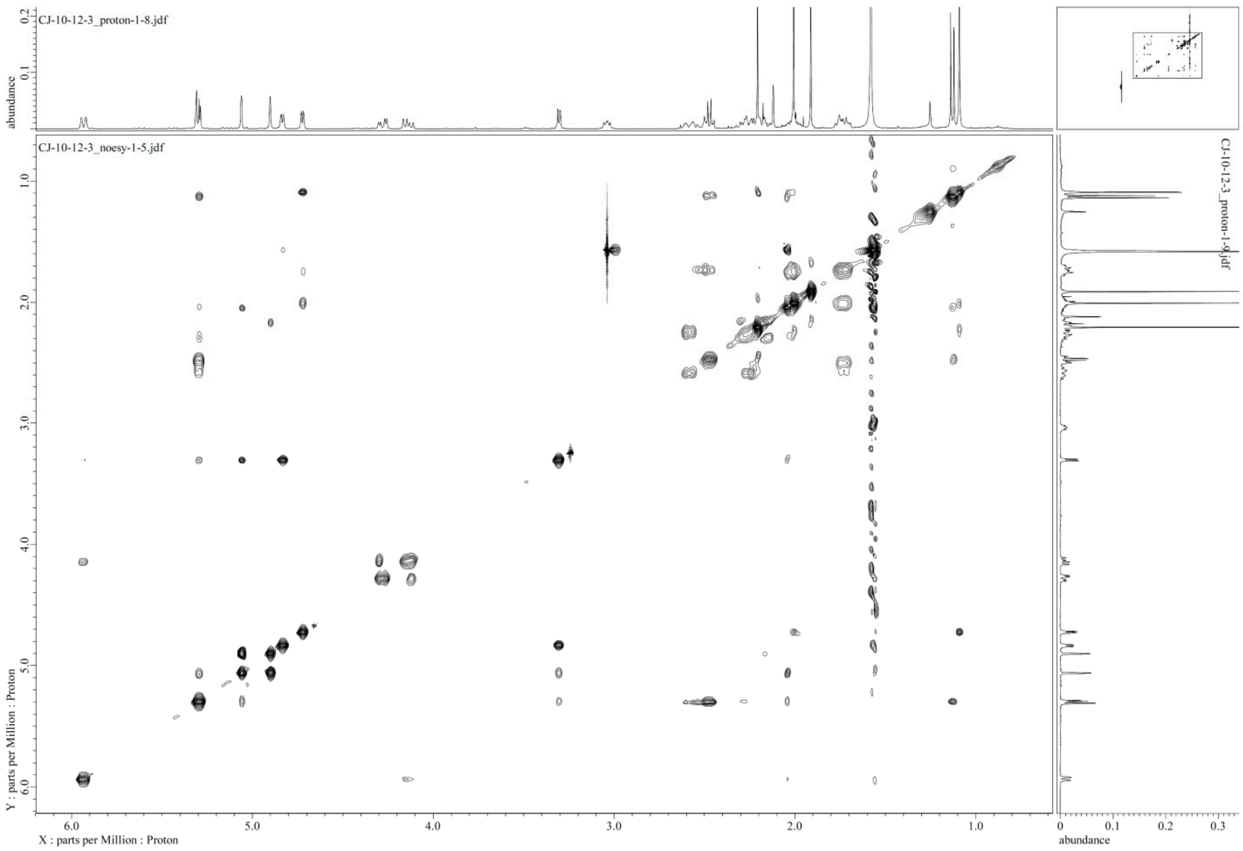

S18. NOESY spectrum of compound **2** in $CDCl_3$.
